# Anorexia nervosa and microbiota: systematic review and critical appraisal

**DOI:** 10.1007/s40519-023-01529-4

**Published:** 2023-02-08

**Authors:** Naomi Garcia, Emilio Gutierrez

**Affiliations:** 1 Departamento de Psicología Clínica Y Psicobiología, Facultad de Psicología, Universidad de Santiago de Compostela, Campus Vida, 15782 Santiago de Compostela, Spain; 2 Unidad Venres Clínicos, Facultad de Psicologıa, Universidad de Santiago de Compostela, Campus Vida, 15782 Santiago de Compostela, Spain

**Keywords:** Anorexia nervosa, Microbiota, Gut-brain axis, Dysbiosis

## Abstract

**Purpose:**

Recent studies have reported a gut microbiota imbalance or *dysbiosis* associated with anorexia nervosa (AN), which has prompted an appraisal of its aetiological role, and the reformulation of AN as a metabo-psychiatric disorder. Thus, the aim of this paper was to critically review the current scientific findings regarding the role of microbiota in anorexia nervosa.

**Methods:**

A systematic study of peer-reviewed literature published in four databases between 2009 and 2022 was conducted according to PRISMA guidelines. Both human and animal studies were included.

**Results:**

A total of 18 studies were included. In animal models, both the preclinical and clinical findings were inconsistent regarding microbiota composition, faecal metabolite concentrations, and the effects of human faecal microbiota transplants.

**Conclusion:**

The methodological limitations, lack of standardisation, and conceptual ambiguity hinder the analysis of microbiota as a key explanatory factor for AN.

**Level of evidence:**

Level I, systematic review.

**Supplementary Information:**

The online version contains supplementary material available at 10.1007/s40519-023-01529-4.

## Introduction

The term intestinal microbiota refers to the set of microorganisms (bacteria, archaea, fungi, and viruses) that coexist in the gut [1]*.* In recent years, intestinal microbiota has become the subject of renewed interest not only in the field of science, but also across society, and industry. Thus, 96.8% of the 53,921 publications in PubMed under the label *“gut microbiota”* were published in the last decade. Moreover, the microbiota has become one of the scientific topics receiving broad coverage in the press [2, 3], and microbiota-based products represent a fast-growing market today, with an estimated 275–400 $ million worldwide [4].

Current research on microbiota has primarily focused on bacteria, most of which belong to a small number of phyla [5]. *Firmicutes* and *Bacteroidetes* represent around 85–90% of the total microbiota, whilst *Actinobacteria*, *Verrucomicrobia,* and *Proteobacteria* appear in smaller proportions [5]. However, this composition is highly variable between individuals and in the same individual over time and can be affected by several factors, with diet being the most researched of all [6]. Both long-term and short-term dietary patterns can produce changes in the structure of microbiota by providing or removing nutrients, and modifying the conditions of the gastrointestinal ecosystem surrounding bacteria e.g., pH, bile acid content, and so forth. [6]. Similarly, antibiotics and psychotropic drugs such as SSRIs have been found to have an impact on the microbiota-gut-brain axis [6], and several studies have shown the composition may vary depending on age, stress, BMI, and physical activity [6], among other factors.

The surge in the interest in microbiota has been motivated by its physiological potential, with an estimated 100 bacterial genes for every human gene [1]. Moreover, gut microorganisms have been considered key players in the gut-brain axis [7], and it has been suggested they may favour communication with the brain via the endocrine, immune, or nervous system [7]. Typically, the prime candidates for establishing this connection are bacterial metabolites (especially SCFAs), neurotransmitters or immune intermediates [6, 7]. Hence, it is reasonable to suggest that an *imbalance* in gut microbiota composition or *dysbiosis* may be linked to the clinical features of several mental health disorders [7]. While the idea that gut microorganisms may affect behaviour is not novel, dating back to Bouchard’s autointoxication theory [8], it is only with the emergence and cheapening of sequencing techniques and bioinformatics that research in the field has developed [7].

Several methods have been applied to the analysis of the microbiota-gut-brain axis [7]. Microbiome studies have employed massive sequencing techniques such as 16S sequencing to compare profiles of microbial communities in individuals with and without pathology [9]. Alternative approaches include Faecal Microbiota Transplantation (FMT) [7] or humanised gnotobiotic rodents, i.e., rodents that have been transplanted with the faecal microbiota of individuals with or without pathology [10]. Thus, research has examined the causal role of microbiota on mental health [7] and, to date, associations for autism, psychotic disorders, anxiety, depression, ADHD, post-traumatic stress disorder and, more recently, eating disorders, including Anorexia Nervosa (AN), have been proposed [7].

AN affects 1% of the population [11] with 90% of cases being women [12]. Its onset is usually associated with puberty or early adulthood and, despite its low prevalence, it has the highest mortality rate among mental disorders [11]. According to the DSM-5, AN is characterized by energy restriction resulting in low body weight, fear of gaining weight, and distorted body image [12]. Two subtypes have been established i.e., restrictive and purgative.

Overall, AN is often associated with physical signs derived from malnutrition such as bradycardia, hypotension, lanugo, hypothermia, and hyperactivity [11]. The latter appears in approximately 70–80% of cases [13] though estimates vary given the lack of consensus in its definition. Furthermore, gastrointestinal symptoms, marked by abdominal pain and discomfort, appear in the literature since the first descriptions [14]. In fact, it has been estimated that 90% of AN cases present some type of gastrointestinal complaint [15].

At present, there is a lack of efficacious treatments for adults with AN [11, 16, 17]. Although international guidelines [18] recommend psychological intervention such as CBT (cognitive behavioural therapy), the MANTRA protocol (Maudsley Model of Anorexia Nervosa Treatment for Adults), and SSCM (Specialist Supportive Clinical Management) as the treatment of choice, a recent meta-analysis conclude that: *“No significant differences between psychological treatment and control condition were found on weight gain, on eating disorder pathology, and on quality of life.*” [16, p.2]. In fact, this could be the outcome of a poorly understood aetiology.

In an attempt to progress, Bulik et al. [19] suggested reformulating/reframing AN as a metabo-psychiatric disorder [19, 20]. This reformulation was grounded in the recent study published by the Eating Disorders Working Group within the framework of the Psychiatric Genomics Consortium (PGC), which concluded the existence of genetic associations between AN and metabolic/anthropometric traits [20].

Under the premise of a metabo-psychiatric disorder, microbiota would emerge as a potential explanatory factor for key features of AN [21]. Therefore, dietary reduction, psychosocial stress, and marked weight loss are considered to contribute to the establishment of a selective intestinal environment for bacterial survival favouring the development of *dysbiosis* [19]. So far, it has been suggested that this might explain low weight gain [19, 22], affective symptomatology [19, 22, 23], altered eating behaviour [23], and even hyperactivity [22]. Thus, modifications of microbiota composition using pro-, prebiotics [22], FMT, or precision nutrition [19] have been proposed as promising therapeutic interventions.

In an effort to determine whether altered composition is more than a mere consequence of malnutrition, several authors have examined the underlying causal mechanisms of microbiota in the aetiology of AN. Intestinal dysbiosis has been suggested to favour tryptophan deficiency by altering serotonergic pathways that increase physical activity [22]. In the same vein, affective symptomatology has been associated with altered gut-brain communication due to increased uremic toxins [22], and reduced SCFAs [21]. Fetissov & Hökfelt [23] define eating disorders as the result of altered communication between microbiota, the immune system, and the neuroendocrine system. Thus, these authors focus on the ClpB protein (caseinolytic peptidase B) generated by members of the *Enterobacteriaceae* family, presenting a mimetic sequence with α-MSH [24]. These authors claim that a dysbiosis involving an increase in these bacteria would cause the segregation of ClpB affecting the aetiology of AN either by (a) a direct effect on the peripheral satiety routes -increasing PYY [25, 26], or centrally through MC4R [27]; or (b) an indirect effect from the formation of anti-ClpB IgG antibodies cross-reactive with α-MSH that would form immune complexes, activating chronically the central MC4R receptor and inducing increased satiety and anxiety, or both. Recently, Frostad [28] has emphasized the role of CCK-4 as a complementary mechanism integrated into this model; malnutrition would cause disruption of the blood–brain barrier, increasing the sensitivity of CCK2 receptors to the CCK-4 peptide generated by enteroendocrine I cells during meals. Thus, anticipatory anxiety would occur that would favour psychological maintenance mechanisms such as the pursuit of thinness and weight overvaluation.

Bearing in mind the above findings on the treatment and aetiological understanding of AN, this work aimed to assess the current scientific findings on the role of intestinal microbiota in the development and maintenance of the disorder in human and animal studies; hence, both human and animal studies were included.

## Method

### Search strategy and study selection

A systematic search was conducted on November 5, 2021 (updated June 13, 2022) of all papers referring to the topic from 2009 to 2022 in the databases PubMed, Web of Science (Wos), PsycINFO, and ScienceDirect. The search consisted of the following combination of terms ((microbiota) OR (microbiome)) OR (dysbiosis)) AND (Anorexia nervosa). PICOS criteria for inclusion and exclusion of studies are shown in Table 1. This review was neither registered nor was a protocol published.

**Table 1:** PICOS study inclusion criteria

Preclinical studies	
(P)opulation	Animal model such as as activity-based anorexia (ABA), or gnotobiotic humanized rodents transplanted with gut microbiota of AN patients (gAN)
(I)ntervention	ABA (food restriction and free access to a running wheel), or faecal microbiota transplantation (FMT) of AN patients
(C)omparison	Mice with ad libitum access to food, without free access to a running wheel as a control for ABA mice. Gnotobiotic mice transplanted with the gut microbiota of normal weight individuals (gNW) as a control for gAN
(O)utcomes	Phenotypical differences between gAN and gNW (behaviour, weight, or others). Microbiota composition in ABA model
** (**S)tudy design	Experimental design
Clinical studies	
(P)opulation	Individuals with AN
(I)ntervention	FMT, treatment with pre-, probiotics or no specified intervention
(C)omparison	NW (normal-weight) control group or no control group
(O)utcomes	The primary outcomes collected were information regarding microbiota analysis in patients with AN (relative or absolute abundance, diversity indices), differences between AN patients and NW controls for these parameters, correlations between microbiota composition and psychopathological parameters, faecal metabolites concentrations (SCFAs, BFCAs or others), effects of FMT, or other applied interventions
(S)tudy design	Randomized or non-randomized, cross-sectional, longitudinal, case series or single-case studies

**Fig. 1:**
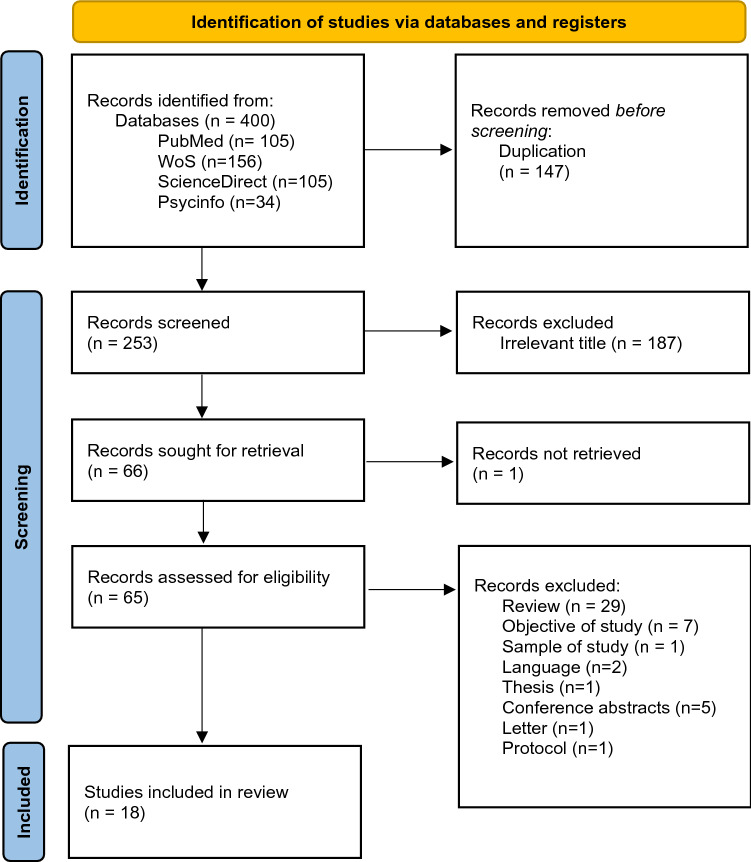
PRISMA flowchart (Page et al., 2021) showing literature search

Only original peer-reviewed published scientific papers were included. The exclusion criteria were as follows: Theses, conference papers, letters to the editor, manuals, literature reviews; studies with authors having conflicts of interest. Articles are written in a language other than English, Spanish, or French. Studies based on populations with anorexia due to other medical conditions. Articles that exclusively evaluated communities of microorganisms other than bacteria or archaea. The search yielded a total of 400 papers, of which 18 papers were finally selected following the guidelines of the PRISMA 2020 protocol [29], as shown in Fig. 1. Moreover, a manual search was performed from the bibliography of the selected paper without adding further references. Article screening was carried out using the Rayyan^®^ web application [30]. Titles and abstracts were independently reviewed by two authors (NG and EG).

### Data extraction and synthesis

Data were extracted to an Excel spreadsheet. The following publication information was sought: sample size and description, intervention or treatment (if applicable), sequencing and analysis methods, gut microbiota outcome data (taxa, diversity) or other outcomes (e.g., metabolite levels, associations between gut microbiota and AN symptoms, FMT outcomes). No statistical analysis was performed due to the heterogeneity of the included studies.

### Risk of bias

The Newscastle-Ottawa scale adapted for cross-sectional studies [31], the SYRCLE’s RoB tool for animal studies [32], and the JBI critical appraisal tools (for case report and case series) [33] were used to assess the potential bias of included studies.

## Results

### Study selection and risk of bias

Of the initial 400 papers, 147 duplicate papers were excluded as shown in Fig. 1. After the primary screening, 187 studies were excluded for not being related to the subject of the study or not being specifically focused on AN. 65 of the remaining 66 were recovered. Finally, a full-text review of these was carried out and 29 reviews were excluded: 2 papers written in a language other than English, French or Spanish; 1 doctoral thesis; 5 conference abstracts; 1 letter to the editor; 1 article with population other than the target; 7 papers with objectives different to the aims of this review, and 1 research protocol.

The quality of the included studies is summarized in Supplementary Tables S1, S2, S3.

### Studies in humans

The present review included five longitudinal studies [34–38], six cross-sectional studies [39–44], two single case reports [45, 46], and a case series design [47] the main features of which are summarized in Table 2.

**Table 2:** Main characteristics of human studies included in the review

Author, year	Study design	Objectives	Sample characteristics	Treatment	Measures	Methods	Main results
Armougom et al., 2009 [39]	Cross-sectional	Identification of specific microbial communities associated with AN, OB, or NW	AN, n = 9 BMI:12.73 ± 1.602 Age: 19–36 OB, n = 20 BMI:47.09 ± 10.66 Age: 17–72 NW, n = 20 BMI:20.68 ± 2.014 Age: 13–68	n/a	Microbiological Assessment Bacteria copy number/ g of faeces	Real-time qPCR of microbiota species (stool sample)	↑ relative abundance of *M. smithii* in AN than in NW
Pfleiderer et al., 2013 [45]	Case Report	Culturomic analysis of AN stool samples	AN, n = 1 BMI: 10.4 Age: 21	n/a	Microbiological Assessment Bacterial identification	Mass spectrometry (MALDI-TOF) Culture Growth	Identification of 11 new bacterial species
Million et al., 2013 [41]	Cross-sectional	Comparison of faecal concentrations of *Escherichia coli, M. smithii, Bifidobacterium animalis,* and *Lactobacillus spp* in OB, OW, NW, and AN	AN, n = 15 BMI:13.5 (11.7–14.6) Age: 27.3 ± 10.8 NW, n = 76 BMI:22.4 (20.7–23.7) Age: 49.5 ± 18.6 OW, n = 38 BMI: 27.1 (25.9–28.6) Age: 54.1 ± 17.8 OB, n = 134 BMI: 40.0 (36.4–46.8) Age: 51.8 ± 14.7	n/a	Microbiological Assessment Prevalence of each bacterial taxonomic group Concentration (log10 copies of DNA / ml)	Real-time PCR of microbiota species (stool sample)	BMI was negatively correlated to *M. smithii, E. coli,* and *B. animalis* BMI was positively correlated to *Lactobacillus reuteri*
Morita et al., 2015 [43]	Cross-sectional	Comparison of intestinal microbiota composition in NW, ANBP, and ANR	ANR, n = 14 BMI: 12.7 ± 1.5 Age: 28.1 ± 10.7 ANBP, n = 11 BMI: 13.0 ± 1.2 Age: 32.5 ± 9.4 NW, n = 21 BMI: 20.5 ± 2.1 Age: 31.5 ± 7.4	n/a	Microbiological Assessment Prevalence of each bacterial taxonomic group log10 cells / g of faeces Biochemical parameters SCFAs	Yakult Intestinal Flora-SCAN (YIF-SCAN®): Reverse transcription-qPCR of microbiota species (stool sample) based on 16S and 23S rRNA analysis Biochemical analysis of blood samples High-performance liquid chromatography (HPLC) of stool samples	↓ bacterial count of *Streptococcus, C. coccoides, C. leptum, B. fragilis,* and *L. plantarum* in AN than in NW ↓ *C. coccoides* in ANR than in NW ↓ *B. fragilis* in ANR and ANBP than in NW ↓ acetic and propionic acid in AN than in NW No significant differences were found in butyrate concentrations between AN and NW
Kleiman et al., 2015 [34]	Longitudinal	Evaluation of changes to intestinal microbiota in AN patients after hospital-based weight restoration Comparison of intestinal microbiota composition in AN and in a normal weight group Assessment of the association between microbial composition, depression, anxiety, and eating disorder psychopathology	AN_0,_ n = 16 BMI: 16.2 ± 1.5 Age:28 ± 11.7 AN_1,_ n = 10 BMI: 17.4 ± 0.9 NW, n = 12 BMI: 21.5 ± 1.9 Age:29.8 ± 11.6	Duration Not specified Type of treatment Weight restoration Other unspecified pharmacological/psychological treatment	Microbiological Assessment Taxa relative abundance α-diversity (species richness, Chao-1 index) and β-diversity (UniFrac distances) Psychological assessment Specific psychopathological characteristics related to eating disorders and, anxious or depressive symptoms	16S rRNA sequencing (V1-V3) (stool sample) BAI, BDI, EDE-Q	↓ genera of *Coriobacteriaceae, Parabacteroidetes* and ↑ genera of *Ruminococcaceae* in AN_1_ than NW ↑ *Bacilli, Coriobacteriales* and ↓ *Clostridiales, Clostridia, Anaerostipes, Faecalibacterium* in AN_0_ than NW ↓ α-diversity in AN_0_ and AN_1_ than NW Significant difference in β-diversity between AN and NW that normalized after treatment α-diversity was negatively associated to EDE-Q total scores, BDI, and subscales scores for shape and weight concern
Mack et al., 2016 [35]	Longitudinal	Comparison of intestinal microbiota composition in AN and NW Evaluation of changes in microbiota composition in post-weight gain and/or normalisation of eating behaviour Assessment of SCFAs profiles (pre- and post-weight gain) dietary intake, and gastrointestinal symptoms	AN_0,_ n = 55 BMI: 15.3 ± 1.4 Age: 23.8 ± 6.8 AN_1,_ n = 44 BMI: 17.7 ± 1.4 NW, n = 55 BMI: 21.6 ± 2.0 Age: 23.7 ± 6,7	Duration 3,5 ± 1,7 months Type of treatment Weight restoration Normalized eating habits Other unspecified pharmacological/psychological treatment	Microbiological Assessment Relative abundance of bacterial taxa Prevalence of bacterial taxa α-diversity (Species richness, Chao-1 index, Shannon index) and β-diversity (unweighted UniFrac distance, Bray–Curtis dissimilarity) SCFAs Gastrointestinal symptoms	16S rRNA sequencing (V4) (stool sample) Gas chromatography Gastro-questionnaire	↓ *Bacteroidetes* to *Firmicutes* ratio in AN_0_ than NW, further decreasing after treatment ↑ *Actinobacteria* in AN_0_ than in NW ↑ *Verrucomicrobia* in AN_0_ than in NW, normalizing after treatment AN_0_ shows ↑ relative abundance of *M. smithii* (but ↓ prevalence), mucin degrading bacteria (*Anaerostipes, Anaerotruncus, Akkermansia), Clostridium* cluster I, XI, XVIII, and *Bifidobacterium* No significant differences in α-diversity between AN and NW Significantly lower β-diversity in the same AN individual at different times (AN_0_-AN_1_) than in different subjects in the respective groups ↑ valerate, iso-butyrate and, BFCAs concentrations in AN_0_ and AN_1_ as compred to NW
Mörkl et al., 2017 [44]	Cross-sectional	Comparison of intestinal microbiota in AN, AT, NW, OW, and OB groups	AN, n = 18 BMI: 15.3 ± 1.3 Age: 22.44 ± 3.20 AT, n = 20 BMI:22.14 ± 1.76 Age: 22.15 ± 3.86 NW, n = 26 BMI:21.89 ± 1.73 Age: 24.93 ± 3.75 OW, n = 22 BMI:26.99 ± 1.13 Age: 25.32 ± 3.98 OB, n = 20 BMI:34.55 ± 4.43 Age: 26.9 ± 6.10	n/a	Microbiological Assessment Relative abundance of bacterial taxa α-diversity (species richness, Chao-1 index, Shannon index) and β-diversity (weighted and unweighted UniFrac distance) Psychological assessment Depressive symptoms Anthropometric assessment (%) body fat Body fat distribution Biochemical Parameters	16S rRNA sequencing (V1-V2) (stool sample) BDI, HAMD BIA, ultrasound measurements	↑ *Coriobacteriaceae* in AN than in NW No significant differences in α- and β-diversity between AN and NW, but significant differences between AN and AT α-diversity was negatively correlated to BDI scores when all groups were included in the analysis
Borgo et al., 2017 [40]	Cross-sectional	Evaluation of the relationship between intestinal microbiota composition, nutritional status, and psychological characteristics in ANR	ANR, n = 15 BMI:13.82 ± 1.80 Age: 25.6 ± 7.97 NW, n = 15 BMI:22.06 ± 2.55 Age: 24.4 ± 5.79	n/a	Microbiological Assessment Relative abundance of bacterial taxa Prevalence of *M. smithii* *M. smithii* copy number α-diversity and β-diversity (OTU-based methods) Psychological assessment General psychopathology, typical cognitive and behavioural characteristics of eating disorders, and anxious or depressive symptoms SCFAs	16S rRNA sequencing (V3-V4) (stool sample) Real-time qPCR for *M. smithii* SCL-90, EDI-2, STAI-Y, BDI-II Gas chromatography	*↓ Firmicutes, Ruminococcus, Roseburia, Clostridium,* and *↑ Proteobacteria, Enterobacteriaceae* in AN than NW ↑ prevalence and absolute abundance of *M. smithii* in AN than NW No significant differences in α- and β-diversity between AN and NW BMI was negatively associated to obsession-compulsion score (SCL-90), state anxiety score (STAI-Y), trait anxiety score (STAI-Y), and BDI total score *Clostridium spp.* was negatively correlated with BDI score ↓ butyrate and propionate levels in AN than in NW No differences in acetate, iso-valerate and iso-butyrate levels between AN and NW Butyrate was negatively correlated to depression and anxiety scores
Kleiman et al., 2017 [47]	Case series	Characterization of daily changes in intestinal microbiota composition and diversity in three acute AN patients during hospital-based renourishment	AN_0,_ n = 3 BMI: 15.6, 17.6,13.7 Age: 25,29,16 AN_1,_ BMI: 20.2, 21.1, 15.4	Duration Between 34 and 73 days Type of treatment Weight restoration Other unspecified pharmacological/psychological treatment	Microbiological Assessment Relative abundance of bacterial taxa α-diversity (Shannon index) and β-diversity (unweighted UniFrac distance) Biochemical parameters	16S rRNA sequencing (V4) (stool sample)	Patient-specific changes in intestinal microbiota composition and diversity during renourishment
Prochazkova et al., 2019 [46]	Case Report	Assessment of intestinal microbiota composition and microbial metabolites post FMT in a woman with AN Evaluation of the effects of FMT on the patient’s psychiatric conditions	AN, n = 1 BMI: 12.36 Age: 37 Healthy donor, n = 1 Age: 67	Type of treatment FMT	Microbiological Assessment Log10 copies 16S rRNA/40 ng gDNA Relative abundance of bacterial and fungal taxa α-diversity (species richness, Chao-1 index, Shannon index) and β-diversity (non-metric multidimensional scaling) Psychological assessment Psychiatric symptoms, family dynamics, and specific eating disorder symptoms SCFAs Metabolites of the tryptophan pathway in the intestine Intestinal permeability I-FABP levels	qPCR 16S rRNA sequencing (V4) and ITS fungal region sequencing (stool sample) EDE-Q, BAI, BDI II NMR, Mass spectrometry	Eating pattern, mood, and gastrointestinal complaints remain unchanged after FMT ↑ absolute abundance of *A. muciniphila* and *M. smithii* at 12 months post-FMT Significant changes in bacterial and fungal composition post-FMT ↑ α-diversity and SCFAs level post-FMT ↓ faecal serotonin level and intestinal permeability post-FMT
Monteleone et al., 2021a [36]	Longitudinal	Evaluation of intestinal microbiota composition in women with AN during weight restoration Evaluation of metabolomic changes in AN pre and post-weight restoration	AN_0,_ n = 21 BMI: 14.6 ± 1.3 Age: 21.7 ± 4.2 AN_1,_ n = 20 BMI: 20.5 ± 0.7 NW, n = 20 BMI: 20.3 ± 1.4 Age: 23.0 ± 3.3 NW_1,_ n = 16	Duration 5 months Type of treatment Nutritional Rehabilitation Enhanced Cognitive Behaviour Therapy 9 patients received SSRI	Microbiological Assessment Relative abundance of bacterial taxa α-diversity (Chao-1 index, Fisher index), and β-diversity (non-metric multidimensional scaling) Psychological assessment General psychopathology, and specific symptoms of eating disorders Metabolomic Analysis	16S rRNA sequencing (V4) (stool sample) EDE-Q, BSI, and SCL-90 gas chromatography–mass spectrometry system	↓ α-diversity in AN_0_ than in NW, normalizing in AN_1_ No significant difference in β-diversity between AN and NW ↑ *Actinobacteria, Coprococcus, Weissella* and ↓ *Bacteroidetes, Firmicutes, Coriobacteriales, Oxalobacteriaceae, Parabacteroides* in AN_0_ than NW ↑ *Firmicutes, Bacteroidetes, Leuconostocaceae* and ↓ *Actinobacteria, Coriobacteriales, Catabacteriaceae, Parabacteroidetes, Collinsella, Catabacter* in AN_1_ than NW Different bacterial taxa were significantly correlated to BMI, EDE-Q total score, and BSI total scores
Monteleone et al., 2021b [42]	Cross- sectional	Comparison of intestinal microbiota composition in NW, ANR, and ANBP Evaluation of the metabolomic profile and its associations with the intestinal microbiota in individuals with ANR and ANBP	ANR, n = 17 BMI: 15.0 ± 1.8 Age: 20.5 ± 3.1 ANBP, n = 6 BMI: 14.7 ± 1.5 Age: 25.2 ± 5.2 NW, n = 20 BMI: 20.3 ± 1.4 Age: 23.0 ± 3.3	n/a	Microbiological Assessment Relative abundance of bacterial taxa α-diversity (Chao-1 index) and β-diversity (non-metric multidimensional scaling) Metabolomic Analysis	16S rRNA (V4) sequencing (stool sample) Gas chromatography–mass spectrometry system	↑ *Verrucomicrobia,* and ↓ *C. coccoides, B. fragilis* in ANR as compared to NW ↓ *Odoribacter, Eubacteriaceae* in ANBP than NW ↓ *Bifidobacterium, Bifidobacteriaceae, Bifidobacteriales* and *↑ Haemophilus, Pasteurellaceae, Pasteurellales* in ANR than ANBP ↓ α-diversity in AN than NW No significant difference in β-diversity between AN and NW Significant differences in metabolomic levels between ANBP, ANR, and NW
Schulz et al., 2021 [38]	Longitudinal	Comparison of intestinal microbiota composition and diversity in adolescents with AN and age-matched NW individuals Evaluation of intestinal microbiota composition and diversity in AN adolescents during pre and post-weight recovery	AN_0,_ n = 19 BMI:15.76 ± 2.03 Age: 15.77 ± 1.94 AN_1,_ n = 19 BMI: 18.8 ± 0.87 NW, n = 20 BMI:20.31 ± 2.35 Age: 16.35 ± 1.11	Duration 4.05 ± 1.39 months Type of treatment Weight restoration Individual and group psychotherapy, parent psychoeducation and training, occupational-, music- and physical therapy	Microbiological Assessment Relative abundance of bacterial taxa α-diversity (Species richness, Chao-1 index, Shannon index) and β-diversity (Bray–Curtis and Jaccard) Psychological assessment Specific characteristics of eating disorders, and anxious or depressive symptoms	16S rRNA sequencing (V1-V2) (stool sample) EDI-2, BDI, SCAS, EDE-Q	No significant differences in α-diversity between AN_0_ and NW ↑ α-diversity in AN_1_ than in NW β-diversity shows significant differences between AN_0_ and NW, no normalizing after treatment ↑ *Anaerostipes* and ↓ *Enterobacteriaceae, Romboutsia* in AN_0_ than in NW ↑ *Firmicutes, Lachnospiraceae, Fusicatenibacter* and ↓ *Enterobacteriaceae, Romboutsia* in AN_1_ than in NW ↑ *Ruminococcaceae, Fusicatenibacter, Lachnospiraceae, Faecalibacterium* and *↓ Bacteroides* in AN_1_ than in AN_0_
Prochazkova et al., 2021 [37]	Longitudinal	Comparison of intestinal microbiota composition and microbial metabolites in AN and NW Comparison of intestinal microbiota composition, neurohormone levels, and SCFAs at hospital admission and discharge Analysis of the composition of the fungal community	ANR_0_ n = 59 BMI: 14 (13.4,15.9) Age: 23 (19,27) ANR_1,_ n = 52 BMI: 17.1 (15.5,18.1) NW, n = 67 BMI: 21.9 (19.9,23.7) Age: 24 (22,28.5)	Duration 51 (28.5, 62.5) days Type of treatment Weight restoration Medication maintenance (32 antidepressants, 16 antipsychotics, 32 others) Unspecified psychological treatment	Microbiological Assessment Relative abundance of bacterial and fungal taxa α-diversity (Chao-1 index, Shannon index, OTU number) and β-diversity (Bray–Curtis and Jaccard) Psychological assessment Specific symptoms of eating disorders Questionnaire addressing hyperactivity, daily habits (sleep, meals), history of stressful events, psychiatric comorbidity, antidepressant, or other medication Hidden eating disorder in healthy controls Anthropometric assessment (%) body fat Biochemical parameters SCFAs Neurohormones	16S rRNA (V3-V4) and fungal ITS2 sequencing (stool sample) EDE-Q, SCOFF BIA NMR, mass spectrometry	BMI increase predicted by several bio-psycho-social factors No significant differences in bacterial classes or genera between ANR_0_, ANR_1_ and NW No significant correlation between α-diversity or bacterial composition and BMI, hyperactivity, disease duration, or EDE-Q scores ↑ α-diversity (only Chao-1 index) in ANR_0_ than in NW and ANR_1_ ↑ β-diversity and core microbiota in ANR_0_ and ANR_1_ than in NW ↑ OTUs related with *Alistipes finegoldi, Alistipes onderdonkii,* and OTUs of *Christensenellaceae, Ruminococcaceae* in ANR_0_ than in NW ↓ OTUs of *Faecalibacterium, Agathobacter, Bacteroides, Blautia, Lachnospira* in ANR_0_ than in NW ↑ *Megapshaera* in ANR_0_ than ANR_1_ Changes in gut microbiota composition related to the length of hospitalization Fungal α-diversity showed no differences between groups ↑ fungal OTUs of *Nakaseomyces* and ↓ *Mucor*, *Naganishia* in ANR_0_ than in NW Differences in predicted metabolic pathways in NW vs. ANR_0_ ↓ GABA, dopamine, butyrate, and acetate levels in ANR_0_ than in NW ↓ serotonine, acetate and propionate levels in ANR_1_ than in NW Different OTUs showed significant associations with propionate, acetate, neurotransmitters, and biochemical or anthropometric parameters

Age and BMI measurements are reported, when required, as mean ± SD. AN (participants with anorexia nervosa), ANR (participants with restrictive AN subtype), ANBP (participants with binge-eating AN subtype), AN_0_ (pre-treatment AN), AN_1_ (post-treatment AN), OB (obesity control group), OW (overweight control group), NW (normal weight control group), SCFAs (short chain fatty acids), qPCR (quantitative polymerase chain reaction), BIA (bioimpedance), OTU (operative taxonomic unit), FMT (faecal microbiota transplantation), NMR (nuclear magnetic resonance), n/a (not applicable)

↓ Significant decrease

↑ Significant increase

One study aimed to analyse the composition of intestinal microbiota in patients with AN [45]. The remaining studies, apart from single-case and case-series studies [45–47], assessed differences in intestinal microbiota between AN and a normal weight control group (NW) adjusted for age and sex. Other studies also compared microbiota composition in an obese group [39, 41, 44], an overweight group [41, 44], and a group of athletes [44]. Seven studies assessed changes in microbiota composition in patients with AN in response to FMT [46], or nutritional rehabilitation [34–38, 47]. Finally, two studies assessed differences in intestinal microbiota composition in AN subtypes in comparison to a control group [42, 43].

Most studies included female participants. In one study a male was included in the AN sample [41], and in another, the sex of participants was not specified [39]. Regarding the AN subtype, most of the studies included patients with both AN subtypes, except for three studies that only included patients with the restrictive subtype [40, 42, 45], one study including patients with the purgative subtype [46], and three studies with unspecified subtype [34, 39, 47]. As for diagnosis, five studies [37–39, 42, 47], used the DSM-5 diagnostic criteria [12], two studies [34, 43] employed the DSM-IV-TR [48], one study [39] used the DSM-IV [49], another [44], the ICD-10 criteria [50], and two studies failed to specify the diagnostic criteria employed [36, 45].

As for symptomatology, an array of instruments had been employed such as SCID-5-CV [36, 42], SCID-I [34, 47], BSI [36, 42], SCL-90 [40], EDI-2 [38, 40], EDE-Q [34, 36–38, 42, 46], EDE [38], SCAS [36, 38, 42], STAI-Y form [40], BDI-II [40, 46], BDI [34, 38, 44], HDRS or HAMD [44], and BAI^1^ [34, 46].

As shown in Supplementary Table S4, the exclusion criteria also differed with two studies using a standard diet for both control and AN groups [36, 42], one monitoring food intake [37], whilst the remaining studies either collected dietary information from structured interviews 24 h diet [35, 44], 3 day self-reports [40], food frequency questionnaires [35], or no type of control was performed [34, 38, 39, 41, 43, 47].

The treatment applied in the studies involved nutritional rehabilitation, except in one case involving FMT [46]. Three studies combined treatment with psychological therapy, either unspecified [35, 38], or cognitive-behavioural therapy [36].

In all studies intestinal microbiota analysis was performed on a faecal sample using real-time qPCR [39, 41], 16S rRNA sequencing [34–38, 40, 42, 44], a combination of both [40], cultivation techniques [45], or reverse transcription qPCR [43]. A variety of primers were used for 16S rRNA sequencing, using both the V1-V2 hypervariable region [38, 44], V1-V3 [34], V3–V4 [37, 40], and V4 only [35, 36, 42, 46, 47]. Furthermore, several studies examined other parameters such as faecal pH [43], short-chain fatty acids (SCFAs) in faecal samples [35, 37, 40, 43], other faecal metabolites [36, 37, 42], intestinal permeability [46], anthropometric parameters [37, 40, 44], metabolic indicators [47], biochemical parameters from a blood sample [40, 43, 44], two studies evaluated fungal communities [41, 46], one study evaluated the severity of functional gastric symptomatology and its post-treatment variation [35], and six studies assessed relationships between microbiota and psychological symptomatology [34, 35, 37, 38, 40, 44].

#### Results of microbiota composition analysis

Two parameters were mainly reported: the relative abundances of different taxonomic groups, and significant differences of these between AN and control groups; as well as the alpha and beta diversity indices. One study reported significant differences at OTU levels [37]. Other studies gathered data on increased *Methanobrevibacter smithii* counts [40], or decreased counts of *Clostridium coccoides, Clostridium leptum, Bacteroides fragilis, Lactobacillus plantarum* in AN patients versus NW [43].

The main results of the comparative analysis on the relative abundance of microbiota in AN patients and NW controls are summarized in Table 3. In spite of certain contradictory results, this analysis showed a lower ratio of the genus *Roseburia*.^2^ In patients with AN [35, 40], a higher relative abundance of *Verrucomicrobia* [35, 42], *Actinobacteria* [35, 36], *Coriobacteriaceae* [34, 44], and *M. smithii* [35, 39], the latter being negatively correlated to BMI [40, 41].

**Table 3:** Significant differences in the relative abundance of intestinal bacterial taxa in individuals with AN as compared to NW controls

Phylum	Family	Genus	Species
*Firmicutes ↓* [36, 40] = [39]* ↑* [35]	*Coriobacteriaceae ↑* [34, 44]	*Ruminococcus ↓* [40]	*M. smithii ↑* [35, 39]
*Proteobacteria ↑* [40]	*Ruminococcaceae ↓* [40]	*Roseburia ↓* [35, 40]	*B. fragilis ↓* [66]
*Bacteroidetes* = [39] *↓* [35, 36]	*Enterobacteriaceae ↑* [40] ↓ [38]	*Clostridium ↓* [40] *Clostridium* cluster *XI* *e Clostridium cluster XVIII ↑* [35]	
*Verrucomicrobia ↑* [35, 42]	*Oxalobacteraceae ↓* [36]	*Lactobacillus* = [39]	
*Actinobacteria ↑* [35, 36]		*Anaerostipes ↑* [35, 38] *↓* [34]	
		*Coprococcus ↑* [36]	
		*Romboutsia ↓* [38]	
		*Faecalibacterium ↓* [34]	
		*Gemmiger ↓* [35]	
		*Anaerotruncus ↑* [35]	
		*Bifidobacterium ↑* [35]	
		*Akkermansia ↑* [35]	
		*Parabacteroides ↓* [36]	
		*Weissella ↑* [36]	

↑ (Significant increase)

↓ (Significant decrease), = (no significant differences)

After treatment studies have shown a lower relative abundance of the *Coriobacteriaceae* family [34, 36], and a higher abundance of *Ruminococcacea*e and the *Firmicute*s phylum as compared to an NW control group [34, 38]. Finally, a lower relative abundance of the genus *Bifidobacterium* was observed, and a higher ratio of *Haemophilus,* and the *Pasteurellaceae* family in the restrictive subtype than in the purgative subtype [42].

Two studies examined the correlations between bacterial ratios and total scores in different psychometric tests [36, 40]. Thus, a negative correlation was observed between the relative abundance of *Clostridium spp* and total BDI scores in patients with AN [40], as well as between different bacterial genera and scores on the BSI, and EDE-Q [36]. However, one study found no correlation with anxiety, depression, or eating disorders symptomatology [38]. One study reported a negative correlation between BMI and a relative abundance of *Bacteroides uniformis* [44].

Ten of fourteen studies reported inconsistent results in microbiota alpha and beta diversity in AN patients in comparison to an NW control group [34–38, 40, 42, 44, 46, 47]. Three studies found lower levels of alpha diversity in AN patients [34, 36, 42], whereas five studies could not replicate these results [35, 37, 38, 40, 44]. As for beta diversity, three studies found significant differences [34, 35, 38], but four did not [36, 40, 42, 44].

Post-treatment comparison of these parameters with respect to NW controls also showed inconsistent results. Thus, one study showed an increase in alpha diversity [38], whilst another found no significant differences [34]. One study found significant differences in beta diversity [38], whereas two did not [34, 36]. Two studies reported negative correlations between alpha diversity and BDI scores [34, 44], but another study only found this correlation for the whole group included in the study [44]. Alpha diversity was negatively correlated to the weight and figure concern subscales of the EDE-Q in one study [34], but this was not replicated in another study [37].

#### Results of analysis of faecal metabolites

A comparative analysis of SCFAs and BFCAs faecal concentrations revealed inconsistent results, as shown in Table 4. One study found a positive correlation between butyrate concentrations and a relative abundance of *Roseburia*, and a greater abundance of valerate and BFCAs concentrations in AN_0_ that was maintained or even increased following treatment with respect to NW [35]. Butyrate concentrations were negatively correlated to anxiety and depressive symptomatology [40]. Three studies analysing other faecal metabolites [36, 37, 42] found lower levels of/ amino-acids and metabolite concentrations from intestinal microbiota in AN patients [36] while establishing differences in the faecal metabolomic profile between ANR and ANP [42].

**Table 4:** Comparison of SCFAS levels (propionate, acetate, butyrate, and valerate), and BFCAS between AN and NW

	PROPIONATE	ACETATE	BUTYRATE	VALERATE	BFCAS
Morita et al., 2015 [43]	↓	↓	=	=	=
Mack et al., 2016 [35]	=	=	=	↑	↑
Borgo et al., 2017 [40]	↓	=	↓		=
Prochazkova et al., 2021 [37]	=	↓	↓		

↑ (Significant increase)

↓ (Significant decrease), = (no significant differences), and blank cell (not measured)

#### Miscellaneous results

One study reported a new species in the gut of AN patients using a culture technique [45], and still another study of an FMT in a case of AN found significant changes in structure, intestinal permeability, diversity, and relative abundances of *A. muciniphila* and *M. smithii* that were unrelated to improved gastrointestinal symptomatology, mood, or eating behaviour [46].

One study evaluating daily changes in intestinal microbiota composition and diversity in three patients observed these changes were specific to each individual [47]. Another study assessing gastric symptomatology and its evolution in response to nutritional rehabilitation found no improvement or even a worsening of symptoms of the upper tract after treatment such as abdominal pain, intestinal noises, and a sensation of incomplete evacuation despite the changes in relative abundances [35]. Finally, another study observed a reduction in faecal GABA and dopamine concentrations in ANR_0_ relative to NW controls [37].

### Animal studies

As shown in Table 5, four studies with different animal models examined the causal role of microbiota in the development of AN [64–67].

**Table 5:** Main characteristics of animal studies included in the review

Author, year	Objectives	Sample characteristics	Intervention/Treatment	Measures	Methods	Main results
Hata et al., 2019 [66]	Comparison of body weight gain and behavioural characteristics between gAN and gNW	AN, *n* = *4* *BMI: 13.7* ± *0.1 kg/m*^*2*^ *Age: 23.0* ± *3.4* NW, *n* = *4* *BMI: 21.6* ± *1.2 kg/m*^*2*^ *Age: 25.3* ± *0.8* Animal model *Female GF BALB/c mice* Sample for microbiological assessment *n*_*gAN*_ = *40* *n*_*gNW*_ = *40* Sample for weight gain and food efficiency analysis *n*_*gAN*_ = *35* *n*_*gNW*_ = *37* Sample for behavioural assessment *n*_*gAN*_ = *35* *n*_*gNW*_ = *37* Sample for analysis of 5-HT and 5-HIIA levels *n*_*gAN*_ = *10* *n*_*gNW*_ = *10*	Experimental intervention Transplantation of faecal microbiota from AN or NW into parental GF mice Probiotic treatment of gAN (*Bacteroides vulgatus,* 5 × 10^8^ UFC/once a week)	Microbiological assessment Relative abundance of bacterial taxa Behavioural analysis time spent in the 12 peripheral subsquares for 20 min number of marbles buried motor activity (total distance travelled for 20 min) Other measures 5-HT and 5-HIAA levels in brain tissue % of body weight change Cumulative food intake Food efficiency	16S rRNA sequencing (V3-V4) (stool sample) Open-field test Marble burying test	↓ Relative abundance of *Bacteroidetes* and *B. fragilis* in AN than in NW ↓ body weight gain in gAN than in gNW ↓ food efficiency in gAN than in gNW Food efficiency was significantly correlated to *Odoribacter* or *Sutterella* gAN spent significantly more time in peripheral subsquares than gNW in an open-field test gAN buried more marbles than gNW in marble burying test ↓ 5-HT levels in gAN brainstem No significant changes in 5-HIAA levels Administration of *B. vulgatus* ↓ nº of buried marbles in gAN mice No significant differences in motor activity between gAN and gNW mice
Trinh et al., 2021 [67]	Analysis of alterations in faecal microbiota in rats using the modified ABA-model with control groups under different starvation and activity conditions	Animal model *Female Wistar rats* C, *n* = *12* CRW, n = *12* RBW, *n* = *12* ABA, *n* = *13*	Experimental conditions CRW RBW ABA	Microbiological assessment Relative abundance of bacterial taxa α-diversity (Species richness, Shannon index) and β-diversity (UniFrac distances) Other measures Body weight (mean g/day) Running-wheel activity (mean distance (km)/ day) Food intake (mean g/day) Histological brain volume GFAP-positive cells per mm^2^ mRNA expression of GFAP	16S rRNA sequencing (V3-V4) (stool sample) Reverse transcription-qPCR Real time-PCR anti-GFAP antibody staining	Chronic food restriction ↑ α-diversity β-diversity in RBW and ABA groups were significantly different from those in the C and CRW groups ↓ *Prevotella, ↑ Odoribacter, Lactobacillus, Akkermansia, Bifidobacterium, Ruminococcus* in animals with reduced bodyweight in comparison to control rats Chronic food restriction had a significant influence on gut microbiota composition
Breton et al., 2021 [64]	Characterization of gut microbiota dysbiosis in an ABA model Correlations between the intestinal bacterial composition and the levels of neuropeptides POMC, or NPY	Animal model *Male C57Bl/6JRj mice* C, *n* = *6–8* LFA, *n* = *6–8* ABA, *n* = *6–8*	Experimental conditions LFA ABA	Microbiological assessment Relative abundance of ASV α-diversity (Species richness and Shannon index) Quantification of neuropeptide expression NPY and POMC mRNA levels Other measures Body weight (mean g/day) Food intake (mean g/day) Running distance for ABA mice (mean distance (km)/ day)	16S rRNA sequencing (V5-V6) in mouse cecum qPCR quantification of mRNA levels	There are no significant differences in α-diversity between C, LFA and ABA mice ↑ relative abundance of *Burkholderiales, Clostridium* clúster XVIII and *Lactobacillus* in C than in ACL and ABA ↑ *C. clocleatum* in ABA and ACL than in C No significant differences in the relative abundance of *Roseburia spp., A. muciniphila* and *M. smithii* between groups *Burkholderiales* was positively correlated to body weight, food intake, and lean mass *Lactobacillales* was negatively correlated to body weight, food intake and lean mass 11 bacterial units were positively correlated to the POMC hypothalamic level 3 bacterial units were negatively correlated to NPY hypothalamic levels All bacterial units were positively correlated to body weight and food intake
Glenny et al., 2021 [65]	Evaluation of the effects AN intestinal microbiota on body composition and weight gain in gnotobiotic mice	AN_0,_ *n* = *4* *BMI: 13.8* ± *0.8 kg/m*^*2*^ *Age: 18.3* ± *0.3* AN_1,_ *n* = *4* *BMI: 18.0* ± *0.9 kg/m*^*2*^ NW, *n* = *4* *BMI: 22.0* ± *0.7 kg/m*^*2*^ *Age: 18.5* ± *0.3* Animal model *Male and female GF C57BL/6 mice* *n*_*gAN0*_ = *50* *n*_*gAN1*_ = *53* *n*_*gNW*_ = *50*	AN treatment Weight restoration Experimental intervention Transplantation of microbiota derived from AN_0,_ AN_1,_ and NW into GF mice (Thawed human stool, oral administration)	Microbiological assessment Relative abundance of bacterial taxa log10 normalized count α-diversity (Shannon index) and β-diversity (non-metric multidimensional scaling, Bray–Curtis dissimilarity) Another measures % change in body composition % change in body weight Average daily food intake (g/day)	16S rRNA sequencing (V4) (human and colonized mice stool samples) qPCR	No relationship between AN-associated intestinal microbiota and changes in body weight, fat mass, lean mass, or daily food consumption No significant differences in α- and β-diversity between gAN_0_ and gAN_1_

Age and BMI measurements are reported, when required, as *mean* ± *SD*. AN_0_ (individuals with anorexia nervosa before treatment), AN_1_ (individuals with anorexia nervosa after treatment), NW (normal weight control group), gAN (gnotobiotic mice reconstituted with gut microbes derived from individuals with AN), gNW (gnotobiotic mice transplanted with gut microbiota of normal weight female subjects), gAN_0_ (gnotobiotic mice transplanted with gut microbiota from AN_0_), gAN_1_ (gnotobiotic mice transplanted with the gut microbiota from AN_1_), C (control animals with ad libitum access to food, without free access to a running wheel), CRW (control animals with ad libitum access to food and free access to a running wheel), RBW (control animals with a 25% reduction in body weight), ABA (experimental group exposed to food restriction and free access to a running wheel), LFA (control group with restricted access to food and without access to a running wheel), GF (germ-free), ASV (amplicon sequence variant), HIAA (5-hydroxyindoleacetic acid), 5-HT (serotonin), POMC (pro-opiomelanocortin), NPY (neuropeptide Y), GFAP (glial fibrillary acidic protein)

↓ Significant decrease

↑ Significant increase

Two of these studies employed germ-free mouse models (*Mus musculus*), C57BL/6 [65], and BALB/c [66]. One study employed C57Bl/6JRj mice [64], while another used Wistar rats [67]. There were also differences regarding the sex of the animals i.e., two studies employed females [66, 67], one study used males [64], and another mixed groups [65].

Two studies performed human faecal microbiota transplants in animal models [65, 66]. One study colonized the same generation orally from frozen faeces [65], while another colonized the parent generation from fresh faeces, using offspring as part of the experiment [66]. The main objectives of these studies were to examine differences in weight gain [65, 66], and behaviour [66] in mice reconstituted with faecal microbiota from AN patients (gAN) as compared to mice with normal weight control microbiota (gNW); and to analyse the relationship between bacterial taxa and weight gain or behaviour [65].

Moreover, two studies evaluated differences in intestinal microbiota composition in animal models under the ABA protocol as compared to control groups in different nutrition and activity conditions [64, 67]. Thus, data are reported for different control groups: a control group without food or activity restrictions (C) [64, 67]; a control group with activity and food restriction [64]; a reduced body weight control group (reduction of food to maintain a 25% lowering in body weight without physical activity), and a control group without food restriction and wheel access (CRW) [67].

In all experiments, microbiota analysis was performed by 16S rRNA sequencing using the V3-V4 region [66, 67], V4 [65] as the primer, or V5–V6 [64]. Furthermore, 5-HT and 5-HIAA concentrations in brain tissue [66], reductions in brain volume and in the number of astrocytes [64], and differences in anxiety levels were assessed using behavioural tests (open field test or marble burying test), as well as the effects of probiotic treatment (*Bacteroides vulgatus*) on behaviour [66].

Faecal microbiota transplant experiments yielded inconsistent results i.e., one study found gAN animals exhibited significantly less weight gain than gNW animals, with longer times in the peripheral quadrants in the open field test, and a higher number of buried marbles, indicating higher levels of anxiety [66], but another study found no significant differences between the two groups [65].

Studies evaluating faecal microbiota in animal models under the ABA protocol showed that in the rat model there were significant differences in composition and diversity between the experimental groups, which were mainly linked to reduced intake rather than hyperactivity [67]. However, no significant differences in alpha diversity were observed in mice, but a higher relative abundance of *Clostridium clocleatum* was found in the ABA model, as well as a positive correlation between the relative abundance of *Burkholderiales* and body weight, food intake, and the percentage of lean mass [65].

## Discussion

In recent years, numerous studies have claimed microbiota is a potential causal explanatory factor for AN. This research is based on the description of an *intestinal dysbiosis* associated to pathology, a deduction underlying most of the human studies included in this review (Supplementary Table S5), which contrasts with the inconsistency in the results obtained (Table 2). The definition of *dysbiotic state* in AN differs from one study to another and has been explained in different ways: due to the immaturity of the techniques, the methodological differences between the studies, and the ambiguity of the term itself.

As indicated by Hooks & O'Malley [68]*, “the broader the definition, the easily it is detected.*” (p.6). The concept of dysbiosis in the current literature poses problems in both precision and consensus and is usually associated with microbiota “imbalance” in terms of differences in the relative abundances and diversity parameters. However, owing to the high inter-individual variability in microbiota composition in humans [5, 9], there is a lack of normative data on what is healthy or normal [68]. Furthermore, the comparison with healthy individuals creates a circular fallacy whereby a pathological process causes dysbiosis which, in turn, leads to a pathological process [4]. Hence, it would be reasonable to ask whether the comparison with a normal weight group does really provide relevant information regarding the aetiology of AN. For microbiota to be regarded as an aetiological factor in AN, it not only has to be different from that of normal weight people but also different from other states of malnutrition.

Furthermore, field studies are still in their infancy, with no clearly established protocols and with major obstacles to overcome in the analyses [69, 70]. Massive sequencing techniques provide data of a compositional nature, collected as relative abundance, which poses a challenge for statistical analysis [69–71]. Thus, an absolute abundance cannot be inferred since the total number of reads per sample provided by the sequencing platforms reflect a technical artifact, unrelated to an actual biological composition [69, 71]. Consequently, the data are not independent of each other, and the increase in the relative abundance of one taxon automatically causes changes in that of others [69, 71, 72]. This implies the need to be prudent in analysing the results. It can be misleading to say that a taxon increases because of a physiological disturbance, without considering a specific reference point [71, 72]. In addition, the probability of false positives, and spurious correlations will increase [69]. At present, even though different analytical solutions have been proposed, the control of these variables has not been completely resolved [70, 71], raising serious doubts regarding the authenticity of the correlations reported in the studies reviewed, as well as the differences obtained between groups.

Moreover, quantitative techniques (qPCR) have been used to obtain absolute data with respect to a specific number of taxa. Though several studies have found a high abundance of *M. smithii*, the biological implications of this finding remain controversial. Thus, it may be involved in constipation often observed in AN, or function as an adaptive factor to low nutrition increasing energy efficiency [73].

In the establishment of causal relationships, that is, in the search for explanatory mechanisms, two methodologies have been used i.e., humanized gnotobiotic animal models and FMT. As for the former, Walter et al. [10] recently pointed out the lack of rigor in these designs*.* A small number of donors are usually used, whose microbiota is transferred to a much larger number of animals, artificially inflating the sample size. This, on the one hand, does not allow representing the high inter-individual variability existing in the composition of the human microbiota and, on the other hand, favours pseudo replication, increasing the probability of false positives [10]. The humanized gnotobiotic animal studies included in this review [65, 66] showed this weakness in design, in addition to divergent results that cast reasonable doubt on the conclusions drawn.

In this review, the FMT treatment was administered to only one patient in one study [46], without positive results in food intake, weight, or affective and gastrointestinal symptoms regardless of the modification in microbiota composition. In the literature, another publication reporting positive results with the same methodology was excluded due to a declared conflict of interest [74].

Bearing in mind the above findings, claims of an intestinal dysbiosis associated to AN are at best speculative given that the current evidence on the proposed theoretical mechanisms is rather scarce [1, 7]. Thus, even though multiple studies have underscored the possible role of SCFAs, the results were highly inconsistent, and failed to support this assumption, as shown in Table 3. As for Fetissov & Hökfelt’s [23] proposal, increases in *Enterobacteriaceae* cannot be substantiated due to its widespread distribution in the human intestine, though it might be a source of antigens even in the absence of any alteration. Thus, this model has two main weaknesses. First, there is the assumption of a common origin for AN, bulimia nervosa, and binge eating disorder [23]. This assumption has been strongly challenged [75, 76], on the grounds of hindering the integration of biological evidence [76], and the homogenization of eating disorders [75] that dilute the importance of fundamental factors for understanding the aetiology of AN, such as malnutrition and its interaction with physical activity [77]. Thus, there is no consistent evidence supporting Fetissov & Hökfelt’s [23] assumption as the data was obtained from heterogeneous samples composed of several of these three categories. Second, correlations between autoantibodies or ClpB and AN have not been established with the most objective AN diagnostic signs, but with subjective symptoms [24, 78, 79], such as the pursuit of thinness. However, as pointed out by Gutiérrez & Carrera [14, 80], this feature is not representative of all AN cases.

Hence, these two shortcomings may explain why IgG autoantibodies against α-MSH have been found in AN patients and normal weight controls [24, 81–83], the high inter-individual variability that has been observed [79, 83], and the inconsistent data regarding the very existence of a greater concentration of this bacteria in AN [24, 79, 83]. Moreover, differences in molecular affinity properties are inconclusive, as sometimes the dissociation rate may be higher [84], or lower [83] in AN than in a normal weight control group.

Moreover, further research on the role of ClpB in peripheral and central satiety pathways is required since its involvement in PYY secretion comes from in vitro studies [26], or from studies with low sample size [25, 26]. Furthermore, one study reported a fragment of the ClpB molecule with an α-MSH homologous sequence with no activity on MC3R and MC4R receptors [85], and no significant differences in concentrations between AN patients and normal weight controls [78].

Finally, further research is required to ascertain if these molecules are transported through the blood–brain barrier, and to determine the mechanisms enabling them to cross the intestinal barrier, as the evidence regarding intestinal hyperpermeability in AN patients is inconsistent [83, 86, 87]. Likewise, the recent integration of the role of CCK-4 in this model [27] is also controversial. The anxiogenic character of this peptide was observed in the 70 s of the XX century and was attributed to an agonistic action on the CCK_2_ brain receptor [88]. However, tetragastrin is a synthetic molecule whose natural endogenous synthesis has not been demonstrated [88, 89]. In addition, the main forms of cholecystokinin (CCK) present in plasma (CCK-58, 33, 22 and 8) [90] have been found in low concentrations, in the range of pMol/L, far from peak in the nanomolar range generated by CCK-4, and it remains to be determined whether they can cross the blood–brain barrier significantly to affect CCK_2_ receptors and have panicogenic activity [88].

A further controversial issue is Sudo’s [22] explanation of hyperactivity in AN, which was inconsistent with the findings of the present review. The animal studies reviewed [64, 67] showed a bacterial composition mainly associated with dietary restriction quite different from the restrictive feeding schedule of the ABA model. Despite observing a lower level of serotonin in the brainstem of the gAN group, Hata et al. [66] reported no significant differences in motor activity. Alterations in the serotonergic pathways associated with AN have been described in the literature, but they have not been established as exclusive to the condition [91]. Serotonin (5-HT) could be involved in increased activity through participation in the aversive motivational system, opposite to the dopaminergic one [92]. However, 5-HT-related interventions tested in the ABA model were partially effective, whereas in humans, they were not [91–93].

### Strength and limits.

The main limitation of this review rest on its qualitative nature, as well as difficulties in interpreting the results given the broad methodological diversity, and the lack of conceptual consensus. Moreover, the dearth of studies evaluating the functionality of microbiota, and the small samples mostly composed of European women, provide a partial view of the issue. Nevertheless, this review seeks to provide insight into the current shortcomings in the field, and to address the explanatory difficulties associated with the assumption of microbiota as a central aetiological agent of AN.

### What we already know on this subject?

In recent years, the etiological role of microbiota in AN has undergone a revival, and studies have underscored the role of intestinal dysbiosis, which has prompted its reconceptualization as a metabo-psychiatric disorder.

### What does this study add?

This review includes clinical and preclinical evidence published in June 2022 regarding the relationship between gut microbiota and AN. The inconsistency in the results failed to substantiate the view of microbiota as an explanatory factor of AN. Shortcomings such as poor consensus, methodological limitations, and ambiguous definitions are inherent to a field that is just starting to emerge/move.

## Conclusions

Owing to the lassitude of the term dysbiosis, and the lack of studies evaluating differences between AN and other cases of malnutrition, the existence of a pathologically specific microbiota composition associated with AN has not been substantiated to date, which undermines its aetiological role. Further research and new protocols are required to advance are our understanding and generate new data to fully elucidate the role of microbiota. However, this accomplishment should not overlook the cautious words of Hanage [94], when he warned, “In pre-scientific times when something happened that people did not understand, they *blamed it on spirits. We must resist the urge to transform our microbial passengers into modern-day phantoms”* (p. 248).

## Supplementary Information

Below is the link to the electronic supplementary material.

Supplementary file1 (DOCX 29 KB)

Supplementary file2 (DOCX 16 KB)

Supplementary file3 (DOCX 14 KB)

Supplementary file4 (DOCX 17 KB)

Supplementary file5 (DOCX 15 KB)

Supplementary file6 (DOCX 19 KB)

## Data Availability

The datasets generated during and/or analysed during the current study are available from the corresponding author on reasonable request.

## Footnotes

1 *Structured Clinical Interview for DSM-5 Disorders—Clinician Version* [51], *Structured Clinical Interview for DSM-IV-TR Axis I Disorders *[52], *Brief Symptom Inventory* [53], *Symptom Checklist 90 *[54], *Eating Disorders Inventory 2* [55], *Eating Disorders Examination Questionnaire* [56], Eating Disorder Examination [57], *Spence Children’s Anxiety Scale *[58], *State Trait Anxiety Inventory Scale* [59], *Beck Depression Inventory-II* [60]*, Beck Depression Inventory* [61]*, Hamilton Depression Rating Scale* [62], *Beck Anxiety Inventory* [63].

2 Prochazkova et al. [37] reported a lower relative abundance of *Agathobacter,* which given the discrepancies in taxonomic annotation, could be comparable to the result with *Roseburia*.
