# Supplementary material for: Anorexia nervosa and microbiota: systematic review and critical appraisal
Source: Eat Weight Disord. 2023 Feb 8;28(1):1. doi: 10.1007/s40519-023-01529-4 (PMC9908645; doi:10.1007/s40519-023-01529-4)
Supplement: Supplementary file 2 — Supplementary file2 (DOCX 16 KB) [file 40519_2023_1529_MOESM2_ESM.docx]

| **Supplementary Table S1.** Risk of bias assessment (Newcastle-Ottawa scale modified for cross-sectional studies) | | | | | | | |
| --- | --- | --- | --- | --- | --- | --- | --- |
|  | Selection (Maximum 5 stars) | | | | Comparability (Maximum 2 stars) | Outcome  (Maximum 3 stars) | |
|  | Representativeness of the sample | Sample size | Non-respondents | Ascertainment of diagnosis | Based on design and analysis^1^ | Microbiological assessment | Statistical test |
| Armougom et al., 2009 [39] |  |  |  | ** |  | * |  |
| Million et al., 2013 [41] |  |  |  | ** |  | ** | * |
| Kleiman et al., 2015 [34] | * |  |  | ** | * | ** | * |
| Morita et al., 2015 [43] | * |  |  | ** | * | ** | * |
| Mack et al., 2016 [35] | * |  |  | ** | * | ** | * |
| Borgo et al., 2017 [40] | * |  |  | ** | ** | ** | * |
| Mörkl et al., 2017 [44] | * |  |  | ** | ** | ** | * |
| Monteleone et al., 2021a [36] | * |  |  | ** | ** | ** | * |
| Monteleone et al., 2021b [42] | * |  |  | ** | ** | ** | * |
| Prochazkova et al., 2021 [37] | * |  |  | ** | ** | ** | * |
| Schulz et al., 2021 [38] | * |  |  | ** | * | ** | * |

*^1^Diet was taken as the main confounding factor. Other confounding factors considered were comorbidities, consumption of probiotics, prebiotics, antibiotics, or psychotropic medication.*
