# Supplementary material for: Anorexia nervosa and microbiota: systematic review and critical appraisal
Source: Eat Weight Disord. 2023 Feb 8;28(1):1. doi: 10.1007/s40519-023-01529-4 (PMC9908645; doi:10.1007/s40519-023-01529-4)
Supplement: Supplementary file 3 — Supplementary file3 (DOCX 14 KB) [file 40519_2023_1529_MOESM3_ESM.docx]

| **Supplementary Table S2.** Risk of bias assessment (JBI critical appraisal tool for case reports and case series) | | | | | | | | | | |
| --- | --- | --- | --- | --- | --- | --- | --- | --- | --- | --- |
| **Case reports** | | | | | | | | | | |
|  | 1 | 2 | 3 | 4 | 5 | 6 | 7 | 8 |  |  |
| Pfeiderer et al., 2013 [45] | No | No | Yes | No | Yes | n/a^1^ | n/a | n/a |  |  |
| Prochazkova et al., 2019 [46] | Yes | Yes | Yes | No | Yes | Yes | Yes | Yes |  |  |
| **Case series** | | | | | | | | | | |
|  | 1 | 2 | 3 | 4 | 5 | 6 | 7 | 8 | 9 | 10 |
| Kleiman et al., 2017 [47] | Yes | Yes | Yes | Yes | Yes | Yes | No | Yes | Yes | Unclear |

^1^Not aplicable (n/a)
