# Supplementary material for: Anorexia nervosa and microbiota: systematic review and critical appraisal
Source: Eat Weight Disord. 2023 Feb 8;28(1):1. doi: 10.1007/s40519-023-01529-4 (PMC9908645; doi:10.1007/s40519-023-01529-4)
Supplement: Supplementary file 4 — Supplementary file4 (DOCX 17 KB) [file 40519_2023_1529_MOESM4_ESM.docx]

| **Supplementary table 3**. Risk of bias assessment (SYRCLE’S RoB Tool for animal studies) | | | | |
| --- | --- | --- | --- | --- |
|  | Hata et al., 2019 [66] | Trinh et al., 2021 [67] | Breton et al., 2021 [64] | Glenny et al., 2021 [65] |
| Random sequence generation  *It was judged as high risk of bias if no information was provided* | **-** | **-** | **-** | **-** |
| Baseline characteristic described | **+** | **+** | **+** | **+** |
| Allocation concealment  *It was judged as high risk of bias if no information was provided* | **-** | **-** | **-** | **-** |
| Random housing  *It was judged as high risk if no information for housing conditions was provided* | **+** | **+** | **+** | **-** |
| Blinding of investigators/caregivers  *It was judged as high risk if no information was provided* | **-** | **-** | **-** | **+** |
| Random outcome assessment | **?** | **?** | **?** | **?** |
| Outcome assessor blinded  *It was judged as unclear risk if no information was provided* | **?** | **+** | **?** | **+** |
| Incomplete outcome data | **-** | **+** | **+** | **+** |
| Selective outcome reporting | **+** | **+** | **+** | **+** |
| Other source of bias  *(unit of analysis error)* | **-** | **+** | **+** | **-** |

Note. (+) Low risk of bias; (-) High risk of bias; (?) Unclear risk of bias
