# Supplementary material for: Anorexia nervosa and microbiota: systematic review and critical appraisal
Source: Eat Weight Disord. 2023 Feb 8;28(1):1. doi: 10.1007/s40519-023-01529-4 (PMC9908645; doi:10.1007/s40519-023-01529-4)
Supplement: Supplementary file 5 — Supplementary file5 (DOCX 15 KB) [file 40519_2023_1529_MOESM5_ESM.docx]

| Supplementary Table S4. Main exclusion criteria of human studies in this review | | |
| --- | --- | --- |
|  | | |
| Exclusion criteria | Studies | |
| Use of antibiotics | | [34, 35, 36, 38, 40, 41, 42, 43, 44, 47, 65] |
| Consumption of probiotics | | [34, 36, 37, 38, 39, 40, 42, 43, 44, 47, 65] |
| Chronic disease (diabetes mellitus, renal failure, cancer) or infections | | [35, 36, 37, 38, 40, 41, 42, 43, 44] |
| Celiac disease | | [34, 36, 38, 40, 42, 44, 47, 65] |
| Inflammatory bowel disease or irritable bowel disease | | [34, 36, 40, 41, 42, 43, 44, 47, 65] |
| Nonsteroidal anti-inflammatory drugs | | [34, 36, 42, 65] |
| Psychotropic medication | | [36, 42] |
