# Supplementary material for: Anorexia nervosa and microbiota: systematic review and critical appraisal
Source: Eat Weight Disord. 2023 Feb 8;28(1):1. doi: 10.1007/s40519-023-01529-4 (PMC9908645; doi:10.1007/s40519-023-01529-4)
Supplement: Supplementary file 6 — Supplementary file6 (DOCX 19 KB) [file 40519_2023_1529_MOESM6_ESM.docx]

**Supplementary Table S5.** Studies describing the presence of gut dysbiosis in AN

| Study | Reference to dysbiosis in AN | Dysbiosis description |
| --- | --- | --- |
| Kleiman et al., 2015 [34] | “We provide evidence of intestinal dysbiosis” (p.2) | ↑ *Bacilli, Coriobacteriales* and ↓ *Clostridiales, Clostridia, Anaerostipes, Faecalibacterium* in AN_0_ than NW  ↓ α-diversity in AN_0_ and AN_1_ than NW  Significant differences in β-diversity between AN and NW that normalizes after treatment |
| Mack et al., 2016 [35] | “the gut microbiota of AN patients is perturbed in comparison to NW participants” (p.2) | ↓ *Bacteroidetes* to *Firmicutes* ratio in AN_0_ than NW, even decreasing after treatment  ↑ *Actinobacteria* in AN_0_ than NW  ↑ *Verrucomicrobia* in AN_0_ than NW, normalizing after treatment  AN_0_ shows ↑ relative abundance of *M. smithii* (but ↓ prevalence), mucin degrading bacteria (*Anaerostipes, Anaerotruncus, Akkermansia), Clostridium* cluster I, XI, XVIII and *Bifidobacterium*  AN and NW showed no significant differences in α-diversity |
| Borgo et al., 2017 [40] | “In conclusion, in the present study a significant dysbiosis was observed in AN patients” (p.13) | *↓ Firmicutes, Ruminococcus, Roseburia, Clostridium,* and *↑ Proteobacteria, Enterobacteriaceae* in AN than NW  ↑ prevalence and absolute abundance of *M. smithii* in AN than NW  No significant differences in α- and β-diversity between AN and NW |

Supplementary Table S5 (continued)

| Mörkl et al., 2017 [44] | “This study provides further evidence of intestinal dysbiosis in AN” (p.1421) | ↑ *Coriobacteriaceae* in AN than in NW.  No significant differences in α- and β-diversity between AN and NW, but significant differences between AN and AT. |
| --- | --- | --- |
| Hata et al., 2019 [66] | “In conclusion, the current results indicate the presence of gut dysbiosis in AN patients.” (p.2450) | ↓ Relative abundance of *Bacteroidetes* and *B. fragilis* in AN than NW |
| Monteleone et al., 2021a [36] | “This finding, although divergent from previous literature data in the types of deranged bacterial taxa, confirm the occurrence of gut dysbiosis in AN” (p.1145) | ↓ α-diversity in AN_0_ than in NW, normalizing in AN_1_  No significant difference in β-diversity between AN and NW  ↑ *Actinobacteria, Coprococcus, Weissella* and ↓ *Bacteroidetes, Firmicutes, Coriobacteriales, Oxalobacteriaceae, Parabacteroides* in AN_0_ than NW |
| Monteleone et al., 2021b [42] | “These findings, although preliminary because of the relatively small sample size, confirm the occurrence of different gut dysbiosis in ANR and ANBP type” (p.11) | **↑** *Verrucomicrobia,* and ↓ *C. coccoides, B. fragilis* in ANR vs. NW  ↓ *Odoribacter, Eubacteriaceae* in ANBP than NW  ↓ α-diversity in AN than in NW.  No significant difference in β-diversity between AN and NW |
| Schulz et al., 2021 [38] | “Our adolescent patients indeed display marked differences compared to HC in this pilot study (...) Interestingly, *this dysbiosis* in adolescents (...)” (p.976) | No significant differences in α-diversity between AN_0_ and NW  Significant differences in β-diversity between AN_0_ and NW  ↑ *Anaerostipes* and ↓ *Enterobacteriaceae, Romboutsia* in AN_0_ vs NW |
